# Predictors and Perceptions of Healthcare Workers Regarding Vaccines Safety in the Initial Phase of COVID-19 Vaccination Drive in Western Part of India: A Regression Analysis

**DOI:** 10.7759/cureus.21267

**Published:** 2022-01-15

**Authors:** Medha Mathur, Anjana Verma, Navgeet Mathur, Yogesh Singhal, Mukul Dixit, Ashish Patyal, Dewesh Kumar, Jignasaben Patani, Suresh Choudhary, Jitendra Hirani

**Affiliations:** 1 Community Medicine, Geetanjali Medical College and Hospital, Udaipur, IND; 2 Community Medicine, Geetanjali Medical College, Udaipur, IND; 3 General Medicine, Geetanjali Medical College and Hospital, Udaipur, IND; 4 Neuro Anaesthesiology, Geetanjali Medical College and Hospital, Udaipur, IND; 5 Community Medicine/Preventive Medicine, Rajendra Institute of Medical Sciences, Ranchi, IND

**Keywords:** india, health professionals, covid-19 vaccine, vaccine hesitancy, vaccine acceptance

## Abstract

Background

The COVID-19 vaccination was launched in a phased manner by the government of India prioritizing healthcare workers. This study assessed the perception of healthcare workers regarding COVID-19 vaccination.

Methods

This cross-sectional study was conducted among healthcare workers vaccinated at a tertiary care center of southern Rajasthan. Logistic regression analysis was used to note the association of perception regarding vaccine safety and other variables.

Results

Out of 3,102, 56.8% were male, and the majority (73.7%) were in the age range of 20-35 years. Out of the total, 80.7% and 73.2% of subjects perceived the vaccine as safe and effective, respectively. The perception regarding the timing of rolling out of vaccine and readiness for COVID-19 appropriate behavior after vaccination was statistically significant (p<0.001). The commonest undesirable effect following vaccination was pain at the injection site. Most of the subjects did not report undesirable effects following vaccination. Logistic regression analysis showed that the involvement in the direct care of COVID-19 patients (OR: 1.58; 95% CI: 1.29, 1.94), the experience of COVID-19 infection in the past (OR: 0.68; 95% CI: 0.50, 0.91), the timing of the rollout of vaccine (OR: 3.60; 95% CI: 3.24, 4.10) showed a significant association with perception of the safety of COVID-19 vaccine.

Conclusions

The vaccine was perceived safe and effective by healthcare workers and reported minimal undesirable effects. The COVID-19 vaccine safety is also dependent on the past COVID-19 infection, involvement in patient care, and time of rollout of the vaccine.

## Introduction

The world encountered a pandemic after reporting unusual respiratory infection cases in Wuhan city of China. There was an urgent need for effective countermeasures against the current emergence and accelerating expansion of coronavirus disease 2019 (COVID-19), caused by severe acute respiratory syndrome coronavirus 2 (SARS-CoV-2) [1]. The induction of herd immunity by mass vaccination has been a very successful strategy for preventing the spread of many infectious diseases, hence protecting the vulnerable masses of the community [2]. Vaccination represents one of the most promising counter-pandemic measures to COVID-19. After introducing the vaccine in India and their availability on a mass scale for healthcare workers, it has been sensed that this group of people may show some vaccine hesitancy [3]. The COVID-19 vaccine was launched by the Government of India on 16th January 2021 for frontline and healthcare workers in a phasic manner [4]. Following the first phase, the vaccination was made available for the elderly (>60 years of age) and individuals between 45 and 59 years with co-morbid conditions. Currently, the vaccination is available for all individuals more than 18 years of age in India, and following the results of ongoing trials, it will also be available for children [5].

It was observed initially that healthcare and frontline workers showed hesitancy and avoided acceptance of the vaccine due to several reasons [6]. The non-acceptance of vaccination by healthcare workers will be considered as a factor for vaccine hesitancy among the general population. The current study was designed to assess the perception of healthcare workers regarding COVID-19 vaccination in terms of its safety, efficacy, and rolling out the strategy. It also aimed to study the beliefs of healthcare workers regarding the need and practice of personal protective measures after vaccination along with the reported side effects by the vaccinees after the COVID-19 vaccination.

## Materials and methods

This cross-sectional study was conducted at COVID-19 vaccination sites of a tertiary care hospital in southern Rajasthan located in the western part of India. The total study duration was of two months. The data collection in the first phase of vaccination was done in the months of January and February 2021 through exit interviews. Seven days after the first dose, the vaccinees were contacted telephonically for reporting of any undesirable effects following immunization. All the healthcare workers visiting the vaccination site for the purpose of COVID-19 vaccination were enrolled for the study by complete enumeration in the first phase of COVID-19 vaccination during the study period. All the healthcare workers vaccinated in the study site and gave consent for participation in the study were included.

Healthcare workers who voluntarily refused to receive the vaccination and those with any contraindication for vaccination (pregnant/ lactating females, active COVID-19 infection, or with a history of recent plasma therapy) were excluded from the study.

A list of all centers designated for vaccination at the tertiary care center was obtained from the nodal officer-in-charge for COVID-19 vaccination of the institute. The principal investigator trained a team of doctors for data collection. All the vaccinees who gave consent for the study were interviewed by trained data collectors using the exit interview technique at the exit point of the vaccination site. All the study subjects after their consent were administered a pre-validated and semi-structured questionnaire via one-to-one interview technique at exit point after completion of the vaccination process, which included registration procedure, waiting, vaccine receiving followed by sitting in the observation area for a minimum of 30 minutes as suggested under the operational guidelines of COVID-19 vaccination [7]. The vaccinees were contacted telephonically to collect data regarding the undesired effects of vaccination post-vaccination up to seven days of duration. The data was collected using a pre-designed and pre-validated tool which was pilot-tested prior to the study. The questionnaire had the demographic details of the study subjects, co-morbidities, their previous experience with COVID-19 infection, and their perception and beliefs regarding the safety and efficacy of the vaccine against the infection. Subsequently, the undesirable effects following vaccination were collected from vaccinees via telephone interview for seven days. Written informed consent was obtained from all study subjects before data collection. Appropriate institutional ethics approval (GU/HREC/2021/1829) was taken from the Institutional Ethics Committee before commencing the study. Data was collected, compiled, and entered in MS Excel software and analyzed using SPSS version 24 (IBM Inc., Armonk, USA). All the categorical variables were presented as frequencies and percentages, and all the continuous variables were shown as mean ± standard deviation (SD). Logistic regression analysis and Chi-square tests were applied to assess the association of various variables studied in the research. P-value <0.05 was considered to be statistically significant.

## Results

A total of 3,102 healthcare workers consented to the study and were included as per the inclusion and exclusion criteria of the study. The number of beneficiaries who were delegated at the centers for the COVID-19 vaccinations was 4,523, and out of those, 3,102 (68.6%) were enrolled for the current study after the consent, while the remaining did not show up for the vaccination or refused to take part in the study. Table 1 shows the demographic profile of study participants.

**Table 1:** Demographic details of healthcare workers (n=3,102) *Students include medical, nursing, physiotherapy undergraduate and postgraduate students. **Others include housekeeping staff, technicians, clerical staff, etc.

Variables	Frequency (percentage)
Age (years)	
<20	415 (13.4)
21-35	2,286 (73.7)
36-50	322 (10.4)
>50	79 (2.5)
Gender	
Male	1,762 (56.8)
Female	1,340 (43.2)
Marital status	
Married	2,179 (70.2)
Unmarried	909 (29.3)
Others	14 (0.5)
Income (Indian rupee)	
<10,000	1,335 (43)
10,000-50,000	862 (27.8)
50,000-100,000	518 (16.7)
>100,000	387 (12.5)
Occupation	
Students*	1,530 (49.3)
Doctors	509 (16.4)
Nursing staff	427 (13.8)
Others **	636 (20.5)

The data related to co-morbidities among study participants stated that 2,853 (92%) people had no underlying condition, while other co-morbidities like diabetes mellitus (n=53; 1.7%), hypertension (n=49; 1.6%), pulmonary issues (n=36; 1.2%), cardiac issues (n=9; 0.3%), neurological (n=10; 0.3%), metabolic (n=29; 0.9%) and other disorders (n=99; 3.2%) including malignancy were prevalent among the study participants.

Table 2 shows that the majority of doctors, nurses, medical students, and other healthcare workers perceived the COVID-19 vaccine as safe (p<0.000), the involvement of healthcare workers in the direct care of COVID-19 affected patients was statistically significant (p<0.000), the experience of being infected with COVID-19 infection ever in the past also showed significant results (p=0.048), the perception of study subjects regarding rollout timing of vaccine and its effectiveness came out to be significant (p<0.000), and the readiness of healthcare workers towards COVID-19 appropriate behavior (wearing a mask, maintain social distancing and hand hygiene practices) were also found to be statistically significant (p<0.001).

**Table 2:** Perception regarding the safety of COVID-19 vaccine among study subjects (n=3,102) *p-value <0.05 A Chi-square test of significance was applied.

Perception of safety of the vaccine	Safe n (%)	Not safe n (%)	Can't say n (%)	p-value
Occupation				
Doctors	383 (12.3)	6 (0.2)	120 (3.8)	<0.001*
Nurses	363 (11.7)	7 (0.2)	57 (1.8)	
Students	1,226 (39.5)	19 (0.6)	285 (9.2)	
Others	530 (17.1)	5 (0.2)	101 (3.2)	
Involved in the direct care of COVID-19 patients				
Yes	848 (27.3)	6 (0.2)	144 (4.6)	<0.001*
No	1,654 (53.3)	22 (1.7)	428 (13.8)	
Experienced COVID-19 infection in the past				
Yes	214 (6.9)	6 (0.2)	61 (2.)	<0.048*
No	2,288 (73.8)	27 (0.9)	506 (16.3)	
Perception regarding the timing of rollout of the vaccine				
Appropriate	2,289 (73.8)	18 (0.6)	452 (14.6)	<0.001*
Delayed	134 (4.3)	6 (0.2)	57 (1.8)	
Pre-mature	79 (2.5)	5 (0.2)	62 (2.0)	
Perception regarding the effectiveness of the vaccine				
Yes	2,095 (65.5)	17 (0.5)	159 (5.1)	<0.001*
No	46 (1.5)	5 (0.2)	6 (0.2)	
Can't say	354 (11.4)	10 (0.3)	410 (13.2)	
Readiness to wear a mask after vaccination				
Yes	2,438 (78.6)	21 (0.7)	541 (17.4)	<0.001*
No	27 (0.9)	6 (0.2)	11 (0.4)	
Can't say	37 (1.2)	7 (0.2)	14 (0.5)	
Readiness to follow social distancing after vaccination				
Yes	2,430 (78.3)	22 (0.7)	536 (17.3)	<0.001*
No	30 (1.0)	5 (0.2)	16 (0.5)	
Can't say	42 (1.4)	6 (0.2)	15 (0.5)	
Readiness to adopt hand hygiene after vaccination				
Yes	2,461 (79.3)	24 (0.8)	560 (18.1)	<0.001*
No	18 (0.6)	5 (0.2)	5 (0.2)	
Can't say	17 (0.5)	5 (0.2)	7 (0.2)	

According to the study subjects, the information regarding the COVID-19 vaccine was obtained from multiple sources like colleagues, friends or family members (n=1,274; 41.1%), social media (n=726; 23.4%), the official website of the Ministry of Health and Family Welfare (n=310; 10%), radio, television or newspaper (n=273; 8.8%), academic forums or webinars (n=355; 11.4%), pharmaceutical company representatives (n=56; 1.8%) and other sources (n=108; 3.5%).

It was found in the study that not all the healthcare workers perceived vaccine as safe and effective against the disease, and only 2,121 (68.4%) got vaccinated by their own choice, while 504 (16.2%) received the vaccination as "everyone was getting vaccinated" and for 428 (13.8%) healthcare workers it was mandatory whereas 49 (1.6%) had other reasons.

Although the acceptance rate was high among healthcare workers for vaccines still, they showed apprehension regarding the vaccine. Out of the total, 611 (19.7%) were worried about the side effects, apprehension for needle prick was observed among 133 (4.3%), 104 (3.4%) feared vaccine-induced COVID-19 like illness, 69 (2.2%) had other issues, while 124 (4%) felt that vaccine may be ineffective. The majority of subjects, i.e., 2,249 (72.5%), reported no apprehension regarding vaccination.

Analysis of the perception regarding the disease prevention through vaccination showed that vaccine will stop the disease occurrence (n=1,202; 38.8%), develop herd immunity (n=1,059; 34.2%), reduce the severity of disease only (n=557; 18%) whereas 127 (4.1%) perceived that vaccine will not change the disease course at all while 156 (5%) stated other reasons.

Nearly 87.8% of healthcare workers had correct knowledge regarding the schedule and recommended duration for the development of immunity (55.3%), and 92.4% of study subjects were willing to get fully vaccinated, i.e., to receive the second dose of vaccine. 

Average duration of vaccination process was 33.90 (±11.34) minutes ranging from 5 to 120 minutes (median=35; interquartile range [IQR]=10; 95% confidence interval [CI] 33.50-34.30). In order to assess the correct time spent in the vaccination process, the duration of the exit interview was not included in the analysis.

Table 3 illustrates the logistic regression analysis of the factors associated with vaccination safety. It includes p-values, odds ratios (ORs), and 95% confidence intervals for each feature using binary logistic regression.

**Table 3:** Logistic regression analysis of variables with perception regarding safety of COVID-19 vaccine ^a^ notes reference category * significant p-value ^#^ includes delayed and hurry ^b^ includes response category "no" + "maybe"

Variable		p-value	OR	95% CI for OR	
				Lower	Upper
Involved in the direct care of COVID-19 patients	Yes	0.01*	1.58	1.29	1.94
	No^a^				
Experienced COVID-19 infection in the past	Yes	0.01*	0.68	0.50	0.91
	No^a^				
Perception regarding the timing of rollout of the vaccine	Appropriate	0.01*	1.54	1.28	1.85
	Not appropriate^#a^				
Perception regarding the effectiveness of vaccine	Yes	0.01*	3.60	3.24	4.01
	No^ab^				
Readiness to wear a mask after vaccination	Yes	0.32	1.18	0.84	1.66
	No^ab^				
Readiness to follow social distancing after vaccination	Yes	0.59	1.09	0.77	1.55
	No^ab^				
Readiness to adopt hand hygiene after vaccination	Yes	0.85	0.96	0.62	1.48
	No^ab^				

The involvement in the direct care of COVID-19 patients (p<0.01; OR: 1.58; 95% CI: 1.29-1.94), experience of COVID-19 infection in the past (p<0.01; OR: 0.68; 95% CI: 0.50-0.91), the timing of rollout of the vaccine (p<0.01; OR: 3.60; 95% CI: 3.24-4.10) showed a significant association with perception of safety of COVID-19 vaccine. Whereas the readiness to follow COVID-19 appropriate behavior was not found to be significantly associated with the perception of the safety of the COVID-19 vaccine.

All 3,102 vaccinated subjects were contacted by telephone to gather data of undesirable effects of vaccination, but only 1,795 (57.9%) responded. Out of 1,795, it was found that 82 (4.6%) received the vaccine for some other ailment in between, so their data was not included in the analysis to avoid any bias. 

**Figure 1:**
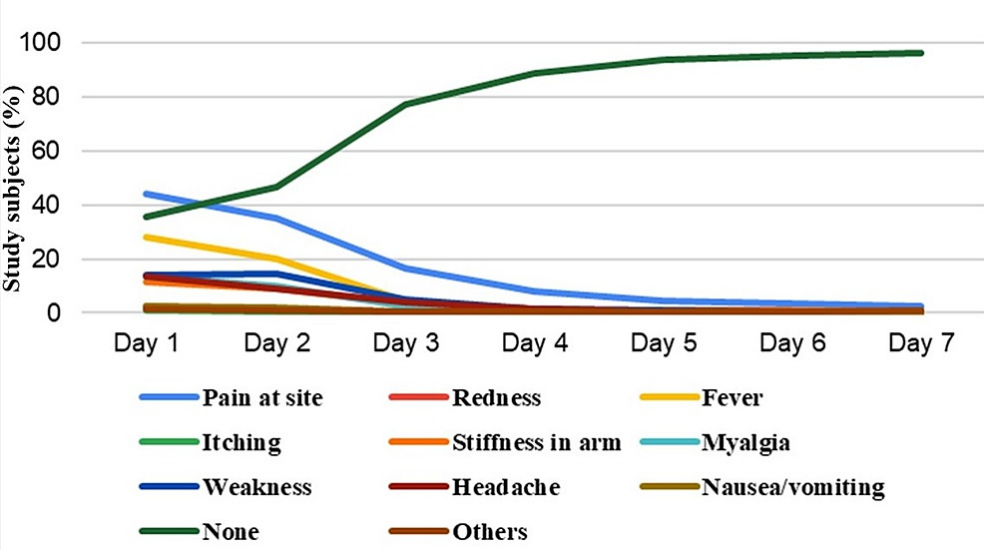
Line diagram showing undesirable effects following COVID-19 vaccination

The undesirable effects among the study participants (n=1,713; see Figure 1) are pain at the vaccination site, redness, fever, itching, stiffness in the arm, myalgia, headache, nausea, and vomiting. The majority of the study participants did not report undesirable effects following immunization, and the frequency of such responses progressed from day one to day seven (see Table 4).

**Table 4:** The undesirable effects following vaccination among study participants (n=1,713) * Responses in the table are not mutually exclusive

Undesirable effect following vaccination*	Day 1 n (%)	Day 2 n (%)	Day 3 n (%)	Day 4 n (%)	Day 5 n (%)	Day 6 n (%)	Day 7 n (%)
Pain at site	754 (43.9)	598 (34.8)	282 (16.4)	134 (7.8)	78 (4.5)	59 (3.4)	40 (2.3)
Redness	42 (2.4)	20 (1.2)	6 (0.3)	5 (0.3)	3 (0.2)	3 (0.2)	3 (0.2)
Fever	481 (28)	347 (20.2)	80 (4.7)	24 (1.4)	8 (0.5)	4 (0.2)	1 (0.1)
Itching	16 (0.9)	12 (0.7)	5 (0.3	2 (0.1)	3 (0.2)	2 (0.1)	3 (0.2)
Stiffness in arm	199 (11.6)	151 (8.8)	58 (3.4)	26 (1.5)	17 (1)	13 (0.8)	9 (0.5)
Myalgia	229 (13.3)	173 (10.1)	43 (2.5)	16 (0.9)	9 (0.5)	10 (0.6)	8 (0.5)
Weakness	240 (14)	246 (14.3)	84 (4.9)	26 (1.5)	14 (0.8)	10 (0.6)	8 (0.5)
Headache	234 (13.6)	155 (9)	66 (3.8)	23 (1.3)	10 (0.6)	5 (0.3)	5 (0.3)
Nausea/vomiting	46 (2.7)	32 (1.9)	8 (0.5)	0 (0)	0 (0)	0 (0)	1 (0.1)
None	610 (35.5)	796 (46.4)	1327 (77.3)	1521 (88.6)	1609 (93.8)	1633 (95.2)	1648 (96)
Others	23 (1.3)	14 (0.8)	7 (0.4)	7 (0.4)	11 (0.6)	11 (0.6)	21 (1.2)

## Discussion

This cross-sectional study was conducted for two months to assess healthcare workers' perception of the safety of the COVID-19 vaccine in the tertiary care institute of southern Rajasthan. A total of 3,102 study subjects were included in the study, who were doctors, nurses, students, and other healthcare workers like housekeeping staff, technicians, and clerical staff.

The male-female ratio among healthcare workers was found to be 1:1.3 in the study, and the maximum study participants (73.7%) subjects were aged from 21 to 35 years. These findings are similar to the study conducted by Kumari et al. regarding qualitative analysis of the perception of Indians and found the male-female ratio of 0.7:1 with a mean age of 36±11 years [8].

The perception regarding the safety of the vaccine among healthcare workers was found to be 80.7% in the current study. These findings agree with a study conducted by Chew et al. on perception and willingness of healthcare workers of the Asian-Pacific region regarding the acceptance of vaccine, which showed that 95% of healthcare workers were willing to get vaccinated and perceived vaccine as safe and with a low harm index according to multivariate analysis. Another study by Kumari et al. also found willingness to get vaccinated [8, 9].

A study on the attitudes of people regarding the COVID-19 vaccine conducted by Praveen et al. via social media analysis reported that fear of health and allergic reactions was the most common; we reported similar findings in the current study where healthcare workers gained information from social media and had apprehension regarding the vaccine [10].

Various studies have been conducted in the Indian context regarding the assessment of knowledge, attitudes, and practices of various strata of the society, including students, the general public, and they concluded that prevention is possible with measures like vaccination, but none of the studies in best of the knowledge of authors has been published with perception regarding the vaccination safety on vaccinated individuals [11-13].

Studies conducted on perception regarding COVID 19 vaccination in India and other countries concluded that among various age groups and strata of the population, the acceptance was an issue due to various reasons like adverse effects, incomplete information or false information, cost, and availability of vaccine [14-18]. 

Apprehensions related to vaccine acceptance resulting in vaccine hesitancy in this study were fear of disease (n=69; 2.2%) and doubt on the efficacy of the vaccine (n=124; 4%), other issues were the side effects (n=611; 19.7%) and worry for needle prick (n=133; 4.3%) whereas 2,249 (72.5%) reported no apprehension regarding vaccination. Other authors reported similar trepidations regarding the COVID-19 vaccination [19].

In the current study, the side effects following vaccination or undesirable effects after vaccination were studied, and it was found that the majority claimed to have no such effects. Still, few subjects reported pain at the vaccination site, redness, fever, itching, stiffness of the arm, myalgia, headache, weakness and nausea vomiting, etc. Similar side effects were declared by vaccine-developing pharmaceutical companies beforehand, and no allergic or life-threatening condition was noted at our center. The mild side effects are acceptable as the vaccine has a protective action against the dreadful disease that has attacked the planet in the form of this pandemic [19,20].

Study limitations include the following: the study was conducted at a single center and on the sample of healthcare workers only due to time and resource constraints. 

## Conclusions

This study reflects the perception of healthcare workers who fought the battle against the COVID-19 pandemic at the forefront regarding acceptance and hesitancy towards its vaccine. The understanding and knowledge are undoubted; still, the beliefs and perceptions regarding the vaccination have been found to overshadow the intellect of healthcare workers when it comes to accepting the vaccination by themselves. The side effects reported by healthcare workers were mild, and none had any serious adverse effects. The knowledge was obtained from various sources, including the official sources of government websites, which helps break the chain of myths and increase acceptability. The vaccine for the general population is available, and the acceptance and hesitancy will be affected directly by the model shown by healthcare workers who presented themselves both for battling the pandemic and accepting the vaccine. 

## References

[1] Sharma O, Sultan AA, Ding H, Triggle CR (2020). A review of the progress and challenges of developing a vaccine for COVID-19. Front Immunol.

[2] Frederiksen LS, Zhang Y, Foged C, Thakur A (2020). The long road toward COVID-19 herd immunity: vaccine platform technologies and mass immunization strategies. Front Immunol.

[3] Caserotti M, Girardi P, Rubaltelli E, Tasso A, Lotto L, Gavaruzzi T (2021). Associations of COVID-19 risk perception with vaccine hesitancy over time for Italian residents. Soc Sci Med.

[4] (2021). Ministry of Health and Family Welfare. https://www.mohfw.gov.in/covid_vaccination/vaccination/index.html.

[5] (2021). COVID-19 vaccine hesitancy worries centre. https://science.thewire.in/health/covid-19-vaccine-hesitancy-worries-centre/.

[6] Ministry of Health and Family Welfare, Government of India (2021). COVID-19 vaccines operational guidelines. https://www.mohfw.gov.in/pdf/COVID19VaccineOG111Chapter16.pdf.

[7] Mandal S, Arinaminpathy N, Bhargava B, Panda S (2021). Responsive and agile vaccination strategies against COVID-19 in India. Lancet Glob Health.

[8] Kumari A, Ranjan P, Chopra S (2021). What Indians think of the COVID-19 vaccine: a qualitative study comprising focus group discussions and thematic analysis. Diabetes Metab Syndr.

[9] Chew NW, Cheong C, Kong G (2021). An Asia-Pacific study on healthcare workers' perceptions of, and willingness to receive, the COVID-19 vaccination. Int J Infect Dis.

[10] Praveen SV, Ittamalla R, Deepak G (2021). Analyzing the attitude of Indian citizens towards COVID-19 vaccine - a text analytics study. Diabetes Metab Syndr.

[11] Gohel KH, Patel PB, Shah PM, Patel JR, Pandit N, Raut A (2021). Knowledge and perceptions about COVID-19 among the medical and allied health science students in India: an online cross-sectional survey. Clin Epidemiol Glob Health.

[12] Sharun K, Rahman CF, Haritha CV, Jose B, Tiwari R, Dhama K (2008). Covid-19 vaccine acceptance: beliefs and barriers associated with vaccination among the general population in india. J Exp Biol Agric Sci.

[13] Vyas H, Goyal R, Meena JK, Mathur M, Yadav A (2020). Knowledge, attitude, and practices in response to COVID-19 pandemic in Indian population. Int J Res Med Sci.

[14] Islam MS, Siddique AB, Akter R, Tasnim R, Sujan MS, Ward PR, Sikder MT (2021). Knowledge, attitudes and perceptions towards COVID-19 vaccinations: a cross-sectional community survey in Bangladesh. medRxiv.

[15] Goruntla N, Chintamani SH, Bhanu P, Samyuktha S, Veerabhadrappa KV, Bhupalam P, Ramaiah JD (2021). Predictors of acceptance and willingness to pay for the COVID-19 vaccine in the general public of India: a health belief model approach. Asian Pac J Trop Med.

[16] Barry M, BaHammam AS (2021). COVID-19 vaccine in the Kingdom of Saudi Arabia: a true operation warp speed. JNSM.

[17] Sallam M (2021). COVID-19 vaccine hesitancy worldwide: a concise systematic review of vaccine acceptance rates. Vaccines.

[18] Tobin EA, Okonofua M, Adeke A, Obi A (2021). Willingness to accept a COVID-19 vaccine in Nigeria: a population-based cross-sectional study. Cent Afr J Public Health.

[19] Akanet A (2021). COVID-19 vaccine availability: what are the side effects?. Br J Gen Pract.

[20] Kaur SP, Gupta V (2020). COVID-19 vaccine: a comprehensive status report. Virus Res.

